# Supplementary figures and images for: Huntington’s disease-associated ankyrin repeat palmitoyl transferases are rate-limiting factors in lysosome formation and fusion
Source: PLoS Genet. 2025 Dec 31;21(12):e1011607. doi: 10.1371/journal.pgen.1011607 (PMC12795455; doi:10.1371/journal.pgen.1011607)

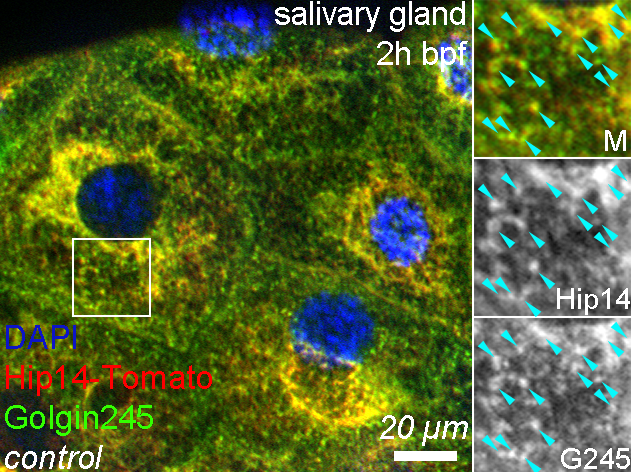

Supplement: S1 Fig — The Hip14-Tomato reporter extensively overlaps (turquoise arrowheads) with the trans-Golgi-specific Golgin-245 marker in control larval salivary gland cells. Insets show 2x magnification of the outlined area, split into channels. Scale bar represents 20 μm. M: merged, G245: Golgin-245, 2h bpf: 2 hours before puparium formation. (TIF) [file pgen.1011607.s001.tif]

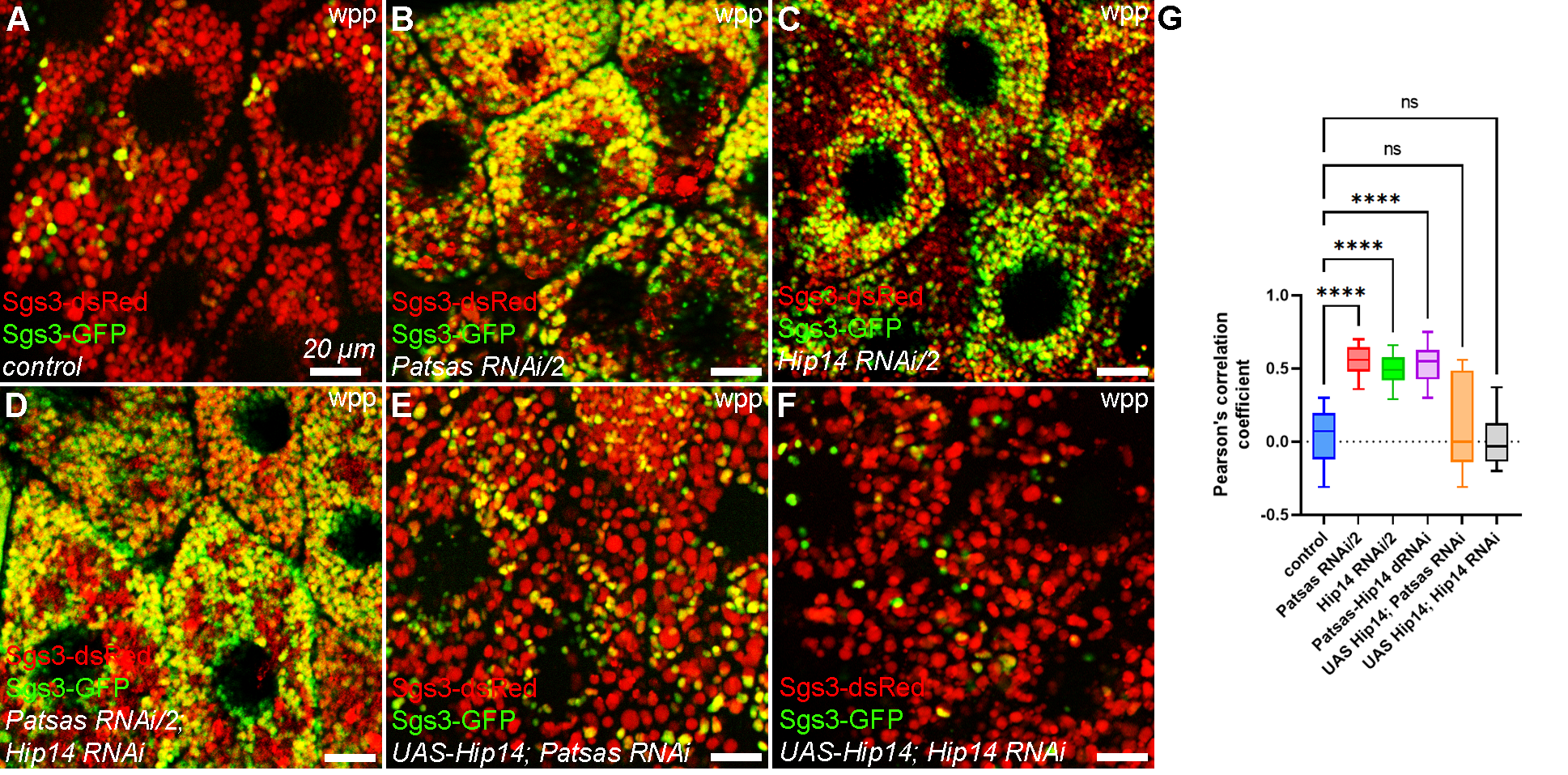

Supplement: S2 Fig — (A) In control salivary gland cells of white prepupae (wpp), most of the Sgs3-GFP signal is quenched in the acidic milieu of the crinosomes; only a few intact double-positive granules can be observed. In contrast, the Sgs3-GFP signal is equally preserved in salivary gland cells expressing independent RNAi transgenes for Patsas (B) and Hip14 (C) or both (D). (E, F) The overexpression of Hip14 could rescue the compromised crinophagic degradation in the absence of either Patsas (E) or Hip14 (F). (G) Quantification of the overlap between the GFP- and dsRed-tagged Sgs3 reporters from n = 25 cells of 5 different larvae, box plots indicate the range of data between the lower and upper quartiles, lines mark the median, ****p < 0.0001, ns p > 0.05. Scale bar represents 20 μm in each panel. wpp: white prepupa. (TIF) [file pgen.1011607.s002.tif]

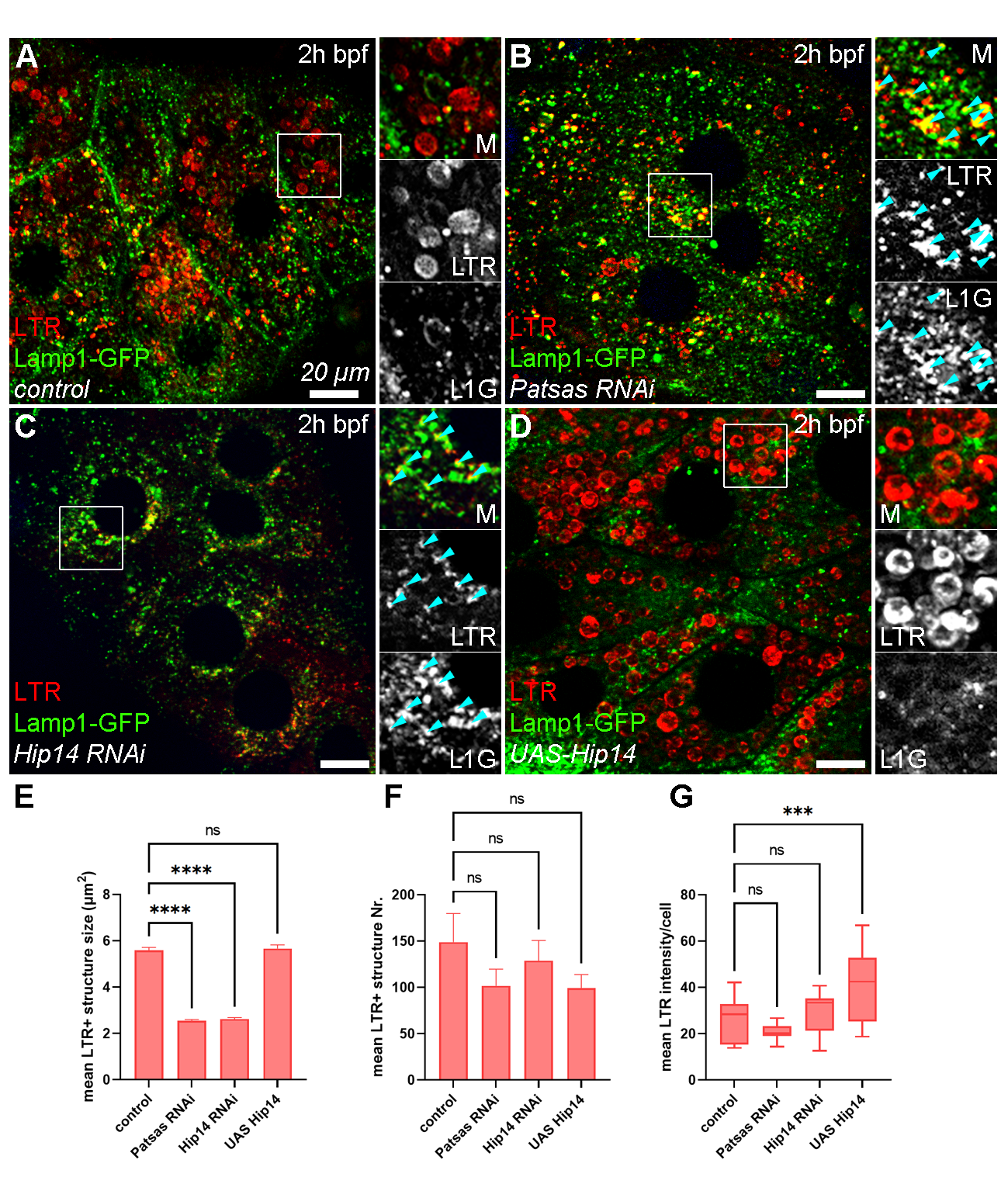

Supplement: S3 Fig — (A) In control salivary gland cells, large LysoTracker Red-positive (LTR+) acidic structures of the size of glue granules appear, in which the GFP signal of the Lamp1-GFP reporter is quenched. (B-C) In the absence of Patsas (B) or Hip14 (C), smaller LTR+ structures appear with retained GFP fluorescence (turquoise arrowheads). (D) In contrast, the overexpression of Hip14 causes the enlargement of LTR+ acidic structures. (E, F) Quantitative assessment of the size (E) and mean number (F) of the LTR+ structures shown in (A-D), n = 2182 (A), n = 2199 (B), n = 3853 (C), n = 2921 (D) LTR+ structure from 5 cells of 5 different larvae, error bars mark ± SEM. (G) Quantification of mean LTR intensities in (A-D), box plots indicate the range of data between the lower and upper quartiles, lines mark the median. ****p < 0.0001, ***p < 0.001, ns p > 0.05. Insets show 2x magnification of the outlined area, split into channels. Scale bar represents 20 μm in each panel. M: merged, L1G: Lamp1-GFP, LTR: LysoTracker Red, 2h bpf: 2 hours before puparium formation. (TIF) [file pgen.1011607.s003.tif]

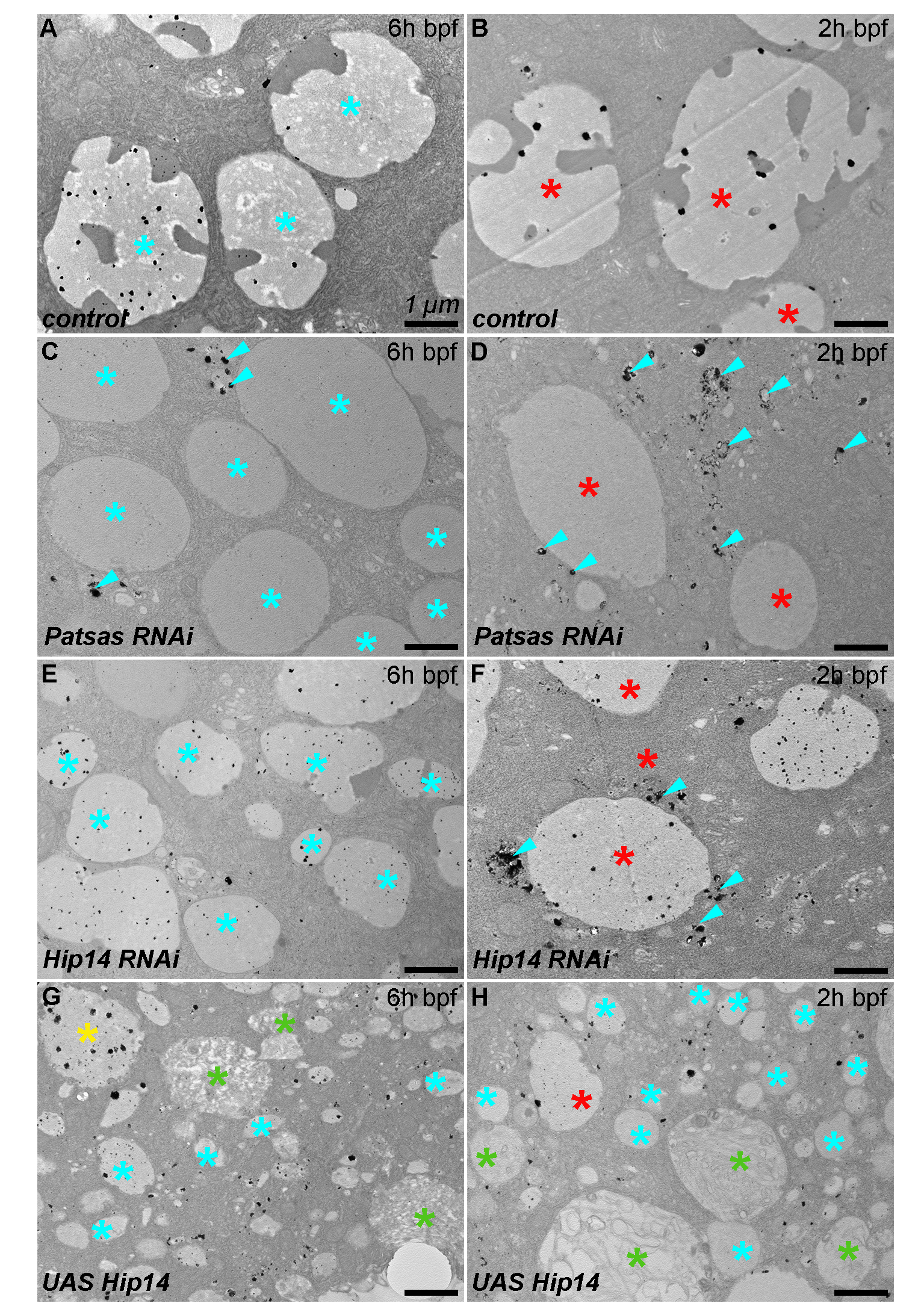

Supplement: S4 Fig — (A) The small immature granules (turquoise star) in control cells of wandering (6h bpf) larvae contain very few acidic phosphatase (AcPase) precipitates (appears as black dots). (B) The mature glue granules (red star) shortly prior to the bulk glue secretion (2h bpf) contain more AcPase. (C) In the absence of Patsas, immature glue granules contain similar level of AcPase signal to the age-matched control, while highly AcPase-reactive lysosomes (turquoise arrowheads) can be observed in the proximity of the granules. (D) 2h bpf stage mature glue granules have a moderate AcPase signal compared to the control, while AcPase-containing (fusion incompetent) lysosomes accumulate among them. (E) The immature glue granules of cells lacking Hip14 show similar AcPase enzyme activity to the control, while the mature glue granules at 2h bpf stage contain slightly less which is accompanied by the appearance of highly AcPase-positive lysosomes at their proximity (F), similar to the Patsas RNAi (D). (G) In contrast, the immature glue granules of Hip14 overexpressing cells contain a lot of AcPase precipitate, with the premature appearance of crinosomes. These crinosomes split into two groups, those with the conventional content containing much (yellow star) and those with an irregular multivesicular morphology (green star) with less AcPase positivity. (H) Later at the 2h bpf stage Hip14 overexpressing cells contain AcPase-positive immature glue granules. Scale bar represents 1 μm in each panel. 6h bpf: 6 hours before puparium formation, 2h bpf: 2 hours before puparium formation. (TIF) [file pgen.1011607.s004.tif]

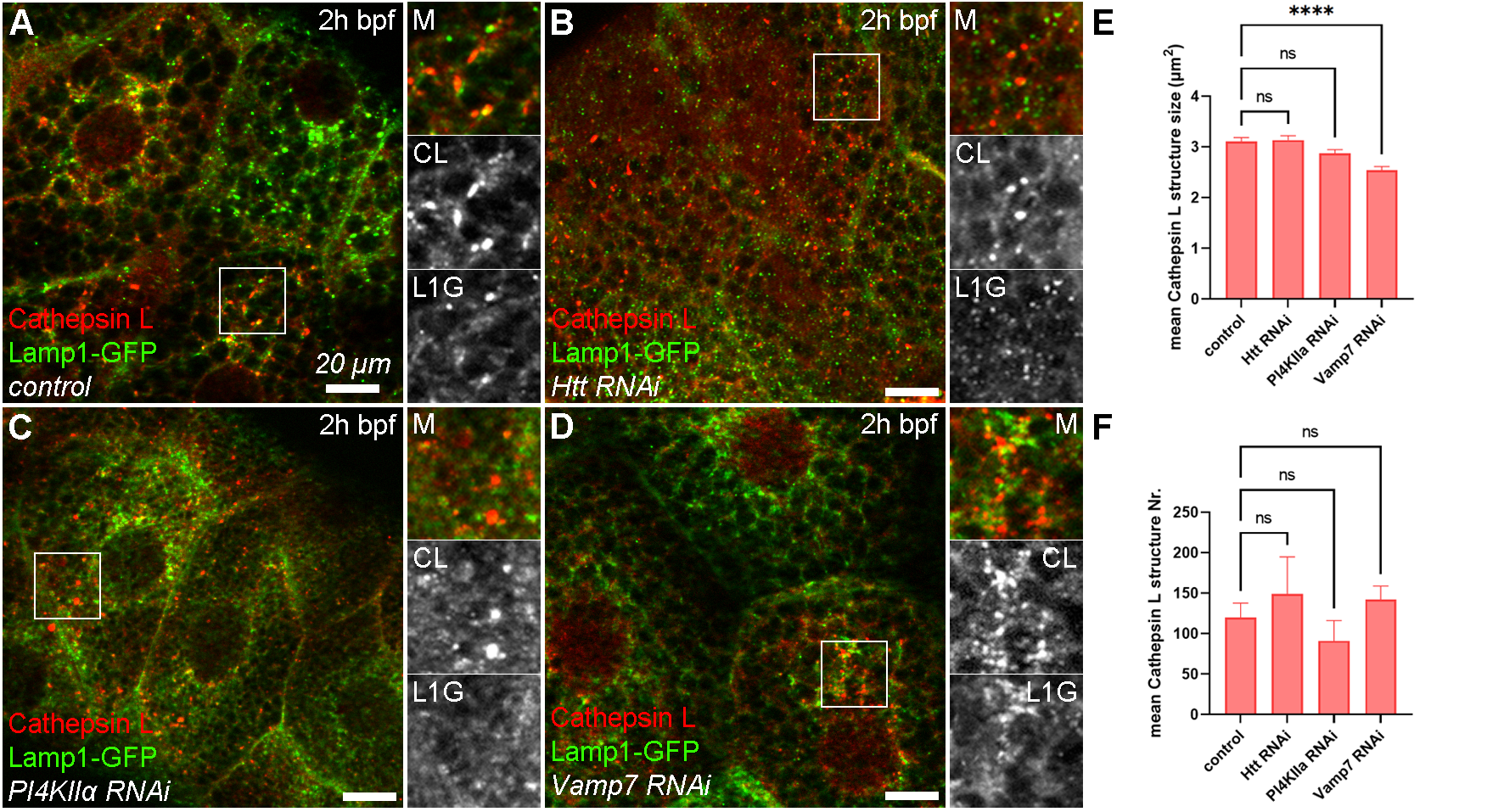

Supplement: S5 Fig — (A) In control salivary gland cells, Cathepsin L partially colocalized with the Lamp1-positive lysosomes. The absence of Htt (B) or PI4KIIα (C) does not impact the size of the Cathepsin L-positive structures. However, the lack of Vamp7 (D) significantly decreases the size of Cathepsin L-positive structures, similarly to Patsas and Hip14. (E, F) Quantification of the size (E) and number (F) of Cathepsin L-positive structures from 5 cells of 5 different larvae, n = 2992 (A), n = 3722 (B), n = 2275 (C), n = 3549 (D) Cathepsin L-positive structures. Error bars mark ± SEM, ****p < 0.0001, ns p > 0.05. Insets show 2x magnification of the outlined area, split into channels. Scale bar represents 20 μm in each panel. M: merged, CL: Cathepsin L, L1G: Lamp1-GFP, 2h bpf: 2 hours before puparium formation. (TIF) [file pgen.1011607.s005.tif]

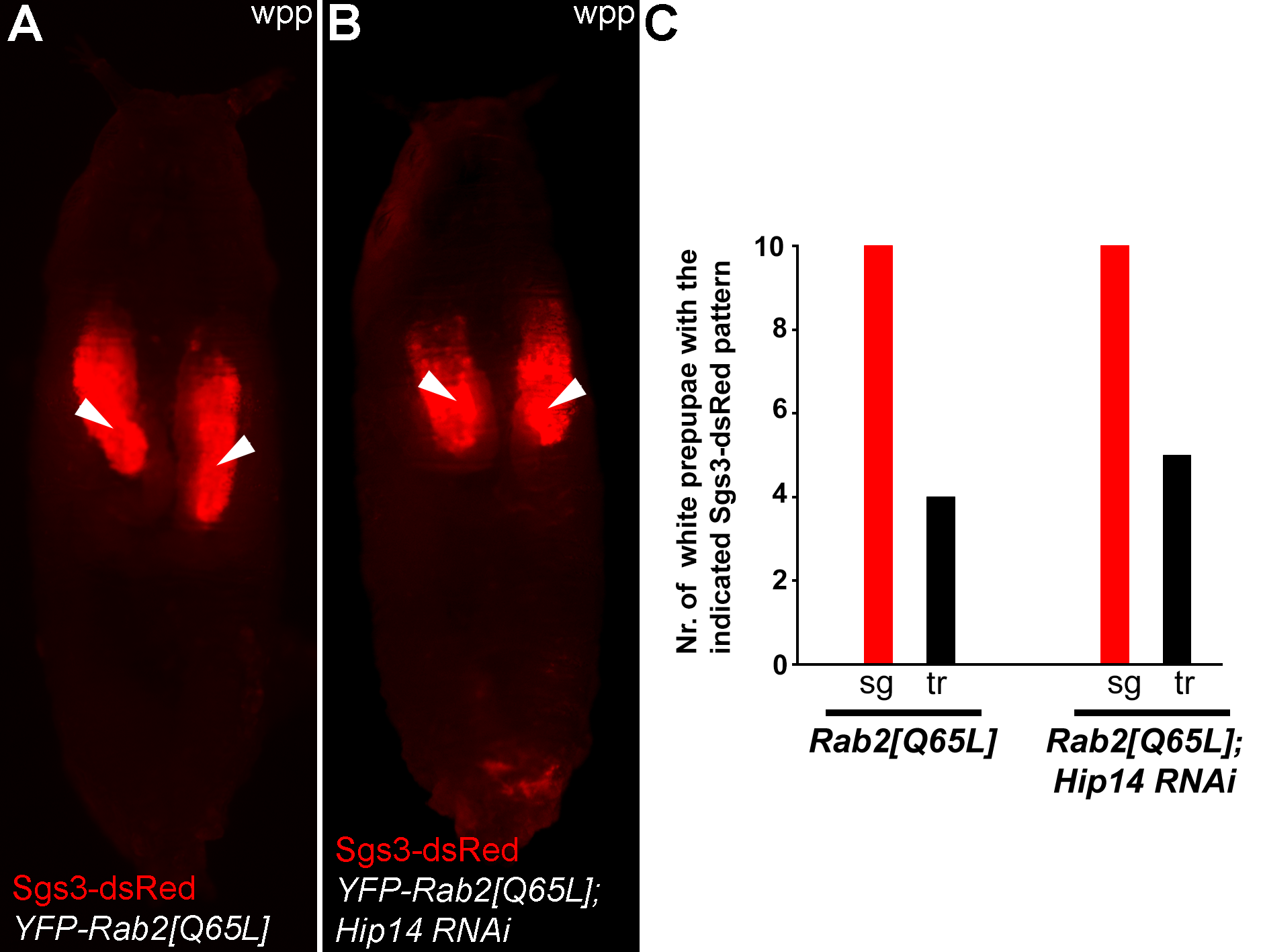

Supplement: S6 Fig — (A) The exocytotic release of Sgs3-dsRed-positive glue granules is compromised in the salivary gland cells of white preupae expressing the constitutively active form of Rab2 small GTPase, thus the glue secretory material is retained in salivary glands (white arrowheads) and absent from traces. (B) The exocytotic release of glue granules is similarly inhibited in salivary gland cells that simultaneously express the constitutively active form of Rab2 and the Hip14 RNAi transgenes. (C) Quantification of the data shown in (A-B), n = 10 different white prepupae per genotype. wpp: white prepupa. (TIF) [file pgen.1011607.s006.tif]

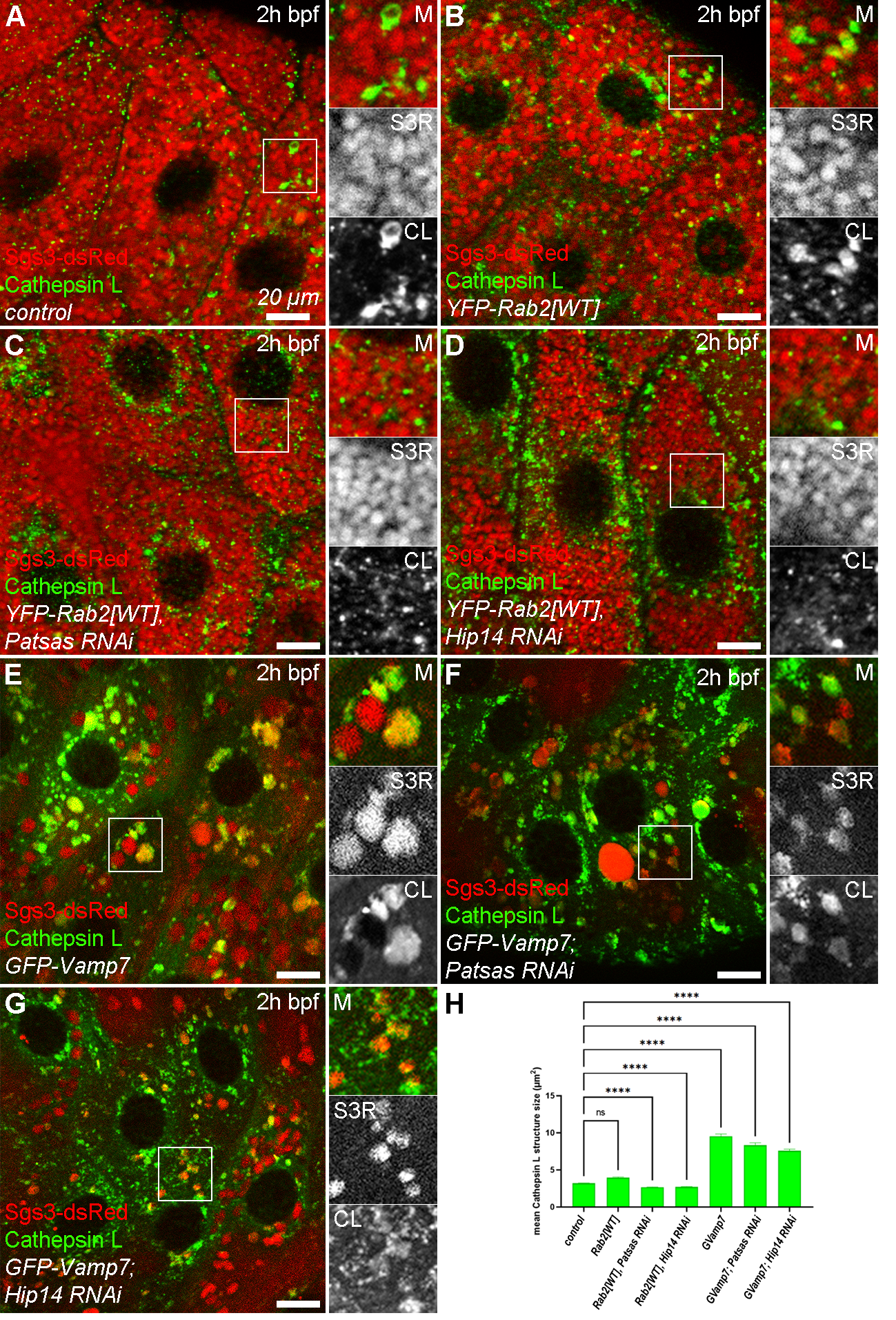

Supplement: S7 Fig — (A-D) In control cells (A), most Cathepsin L-positive lysosomes are located among the secretory granules and overexpression of wild-type Rab2 in a control background (B) does not alter the size of Cathepsin L-positive structures, which remain smaller in the absence of Patsas (C) or Hip14 RNAi (D). (E-G) In contrast, the overexpression of Vamp7 (E) promotes fusion between Cathepsin L-positive lysosomes and maturing granules, thereby increasing their size, even in the absence of Patsas (F) and Hip14 (G). (H) Quantification of the size of Cathepsin L-positive structures shown in (A-G), n = 6165 (A), n = 4260 (B), n = 4277 (C), n = 3076 (D) n = 1110 (E), n = 613 (F), n = 759 (G) Cathepsin L-positive structures from 5 cells of 5 different larvae. Error bars mark ± SEM, **** p < 0.0001, ns p > 0.05. Insets show 2x magnification of the outlined area, split into channels. Scale bar represents 20μm in each panel. M: merged, CL: Cathepsin L, S3R: Sgs3-dsRed, 2h bpf: 2 hours before puparium formation. (TIF) [file pgen.1011607.s007.tif]

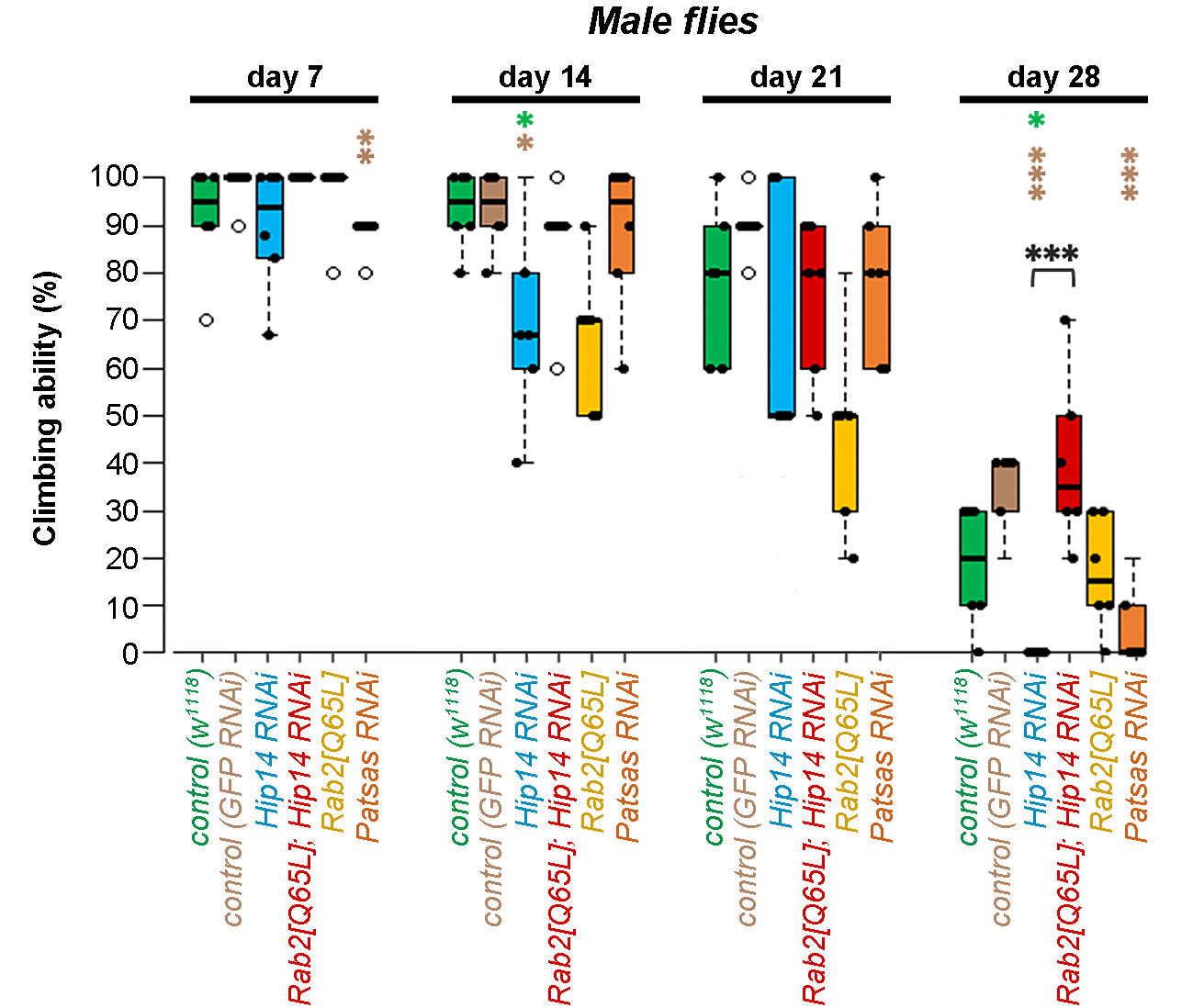

Supplement: S8 Fig — In contrast to the control (w1118 and GFP RNAi) flies, the brain-specific knock-down of Hip14 causes a significant decline in neuromotor performance, especially in 28 days-old, aged males. However, this impaired climbing performance can be improved by the expression of constitutively active form of Rab2 small GTPase. The brain-specific loss of Patsas reduces the climbing performance, particularly in aged males, similar to Hip14. Black dots represent one measurement, circles indicate outliers, boxes represent the typical 50% of the climbed adults, the lines show the median and whiskers present the upper and lower quartiles, n = 20 flies per genotype. Significant differences are indicated by *** p < 0.001, ** p < 0.01, * p < 0.05, green and brown *-s represent significant difference compared to control (w1118) and control (GFP RNAi) respectively. Otherwise, significant differences between two genotypes are represented by black *-s over the clasps. (TIF) [file pgen.1011607.s008.tif]
