# Supplementary material for: Huntington’s disease-associated ankyrin repeat palmitoyl transferases are rate-limiting factors in lysosome formation and fusion
Source: PLoS Genet. 2025 Dec 31;21(12):e1011607. doi: 10.1371/journal.pgen.1011607 (PMC12795455; doi:10.1371/journal.pgen.1011607)
Supplement: Attachment — Submitted filename: HIP14.doc [file pgen.1011607.s010.doc]

Post-translational modifications (PTMs) of proteins play important roles in regulating the subcellular localization, function, and stability of proteins, diversifying the spectrum of proteosome and involving in multiple biological processes. Palmitoylation is one of the PTMs, catalyzed by DHHC proteins in the ER and golgi apparatus. It has been suggested that accumulation of mutant Huntingtin proteins can reduce the activity of DHHCs like HIP14 and HIP14L (Patsas). However, how the events are triggered and the subsequent of these PATs loss of function in the pathogenesis of Huntingtin disease remains largely unclear. In this manuscript, the authors used the salivary gland (SG) of <*Drosophila>* to explore the roles of HIP14 and HIP14L in the exocytosis and lysosomal degradation of glue granules, and later examined the phenotypes of HIP14/L in neurons to implicate the possible roles JIP14/L played in the pathogenesis of Huntingtin disease.

Although the authors found that HIP14/L may participate in the lysosomal degradation of un-secreted glue granules in the SG, directly or indirectly, and affect the neuronal functions, the manuscript is basically describing the phenotypes. No mechanism of the function of HIP14/L is attempted to be addressed at all. As the enzyme responsible for protein palmitoylation, how do HIP14/L function in this process? Which substrates are they palmitoylated? Do they function simultaneously in the secretion of glue granules and lysosomal components or they involve in the degradation of non-secreted glue granules alone? Do they function similarly in neurons as they do in the SG? Considering theses important unanswered questions, I would not recommend the publication of this manuscript, unless these questions are properly addressed.

Other concerns:

1. As HIP14 and HIP14L are homolog, are they equally required or differentially in the degradation of unsecreted glue granules? If they play redundant role in this process, why depleting one of them causes defects? If they function differentially, why similar defects are observed when they are knocked down?
2. The authors showed that both the degradation of un-secreted glue granules and secretion (exocytose of lysosomal components to the PM) are affected, how are the degradation of these glue granules processed?
3. in Figure 1, the authors indicated that the stage of development is prepupal stage, this is very confusing. The authors should indicate the defined time point of developmental stage, such as indicated in Figure 2, 2 hours before pupation.
4. Again in Figure 1, as they examined the SG at the same developmental stage and the same marker (Sgs3-dsRed) is used, why there are dramatic differences in the number of Sgs3-red granules between Fig. 1A-C and Fig. 1E-G?
5. for the forced secretion/transport of glue granules by ectopic expression of constitutively active Rab2, as Rab2CA promotes the secretion of glue granules and fusion of un-secreted granules with lysosome, why there are so many glue granules observed in Rab2CA-expressing cells? The authors also found that ectopic Rab2CA overrides the defects observed in HIP14/L knockdown cells, how this happens?
6. the transition from the SG to the neurons/brain is not supported by the same mechanism as assumed by the authors.
