# Supplementary material for: Huntington’s disease-associated ankyrin repeat palmitoyl transferases are rate-limiting factors in lysosome formation and fusion
Source: PLoS Genet. 2025 Dec 31;21(12):e1011607. doi: 10.1371/journal.pgen.1011607 (PMC12795455; doi:10.1371/journal.pgen.1011607)
Supplement: Attachment — Submitted filename: Response to reviewers.docx [file pgen.1011607.s011.docx]

**Response to reviewers**

We thank the expert reviewers for their useful comments and suggestions on how to improve our manuscript. Accordingly, we have addressed all of their concerns and carried out new experiments.

Following your comments and suggestions we did additional quantifications on the already existing data and also carried out new experiments, which led to the extension of the already existing figures, and we designed 4 new main and 6+1 supplementary figures (one of them, **Supplementary Fig S3** in the revised version corresponds to the **former** **Fig 3** main figure of the original version of the manuscript). We would also like to inform you that in addition to addressing your comments, we performed several further changes on the figures and the text. This resulted in the renaming of the former main figures and the main text without any substantial change in the logic of the experiments and the story. We also rearranged the order of some figure panels, without changing their data content. We only changed panels when it was claimed by one of your comments. We also corrected several typos and changed the blue pseudocolor to green in microscopy images in **Fig 7** (corresponds to the **former Fig 6**) for better visibility. Finally, we generated a new summary figure (**Fig 10**) to summarize our suggested model how PATs are affecting the fusion of secretory granules (SG) and lysosomes.

We summarized all changes in Figure panels in the following table:

| panel in Figure in revised version | corresponding panel in Figure in the original version | panel in Figure in revised version | panel in Figure in the original version |
| --- | --- | --- | --- |
| 1B | 1B new image | 7A | 6A recolored |
| 1D | 1E | 7B | 6B recolored |
| 1E | 1F | 7C | 6C recolored |
| 1F | 1G | 7D | 6D recolored |
| 1G | - | 7E | 6E recolored |
| 1H | 1D | 7F | - |
| 1I | - | 7G | - |
| 1J | - | 7H | 6F |
| 1K | 1H | 7I | 6G |
| 2B | 2B new image | 7J | 6H |
| 2D | - | 7K | 6I |
| 2E | 2D double RNAi data | 7L | 6J |
| 2F | - | 7M | - |
| 2G | - | Figure 8 | Figure7 significantly modified |
| 3A-3I | - | Figure 9 | Figure7 significantly modified |
| 4C | 4C new image | S3A | 3A new image |
| 6A | 5A | S3B | 3B added arrowheads |
| 6B | 5B | S3C | 3C added arrowheads |
| 6C | 5C | S3D | 3D |
| 6D | 5D | S3E | 3E recalculated |
| 6E | 5E | Figure S4 | Figure S1 |
| 6F-6L | - |  |  |

Please see below for our point by point response (written in red) to your comments:

**Reviewer #1:** Győző Szenci et al. present novel and compelling evidence that the palmitoyl-transferases Patsas and Hip14 are key regulators of the interplay between lysosomes and the secretory pathway. The authors show that both enzymes support efficient crinophagic degradation by promoting fusion of residual glue granules with PI3P-positive endosomes, a step required for crinosome formation. Perturbing either HIP14 or Patsas impairs lysosomal maturation and fusion, leading to glue-granule accumulation in the Drosophila salivary gland and to climbing defects in adult flies. These findings suggest that Patsas and Hip14 act as rate-limiting factors in lysosome biogenesis/fusion and may protect against Huntington-disease-like phenotypes. However, the precise mechanism by which HIP14 and Patsas control lysosomal dynamics, especially in neurons, remains unclear. I therefore have several questions and suggestions, detailed below.

Thank you for the positive evaluation of our work!

Major
1. Each gene is targeted with a single RNAi construct, which raises concerns about potential off-target effects or variable knockdown efficiency. Including an independent RNAi line would greatly strengthen the conclusion that the observed phenotypes are specific and reproducible.

Thank you very much for this comment. We carried out the same test with independent RNAi lines, which we refer to as *Hip14 RNAi/2* and *Patsas RNAi/2* in the text and figure legends. Importantly, all experiments with these independent RNAi lines resulted in the same phenotype as the original ones (please see **Supplementary Fig S2A-C, G),** indicating that the RNAis we used were efficient and their effect is reproducible.

2. Are HIP14 and Patsas localized and functional in the same way in neuronal tissue as in salivary-gland cells?

Thank you for this comment. The mammalian orthologs of Hip14 and Patsas, HIP14 and HIP14L were shown to be Golgi-resident enzymes (PMID: 18794299, PMID: 19299482). Moreover, Golgi localization of Drosophila Hip14 in adult brain is also well established and demonstrated by at least two labs (PMID: 39475475 and 18794299), and Golgi localization of both Patsas and Hip14 was already demonstrated in cultured Drosophila S2 cells (PMID: 18719403). One of these studies used the same *Hip14-Tomato* reporter which we also obtained, and applied for immunolabellings on salivary gland, where we observed extensive colocalization between this Hip14-Tomato reporter and anti-Golgin245 Golgi marker (see **Supplementary Fig S1**). Unfortunately, we could not obtain a transgenic fly line carrying a Patsas reporter, hence we could not test the localization of this protein. However, based on our findings with the Hip14 reporter we strongly assume that the localization of Patsas may also be highly conserved. Moreover, the above-mentioned studies carried out in in other species and cultured Drosophila cells that showed that both ankyrin-repeat PAT-s localize to the Golgi further strengthen this assumption.

We found that ANK PATs have similar effect on lysosome acidification and function both in larval salivary glands and adult brains. By using a combination of pH-sensitive reporter Lamp1-GFP, whose signal is quenched in acidic lysosome lumen and LysoTracker Red vital dye (please also see detailed description of the use of these two markers in our response for **comment 12 of Reviewer 2**), we demonstrated that loss of Patsas and Hip14 reduces the acidification of lysosomes and increases the colocalization of these two markers in both tissues. In contrast, these two markers barely colocalize in control samples from both tissue types (see **Fig 8F-H** and **Supplementary Fig S3A-C**). Moreover, perturbation of crinophagic flux indicated by the loss of the pH-dependent quenching of Sgs3-GFP signal in Patsas and Hip14 RNAi in salivary glands of white prepupae further supports this finding (see **Fig 1A-C, G, H** and **Supplementary Fig S2A-D, G**). Additionally, we presented that loss of Patsas or Hip14 severely perturbs the morphology of Cathepsin L positive lysosomal compartment. Although this perturbation caused changes in lysosome size, the direction of these were not the same in these two tissues (decreased in salivary gland 2h bpf vs. increased in adult brain) (see **Fig 6A-C, E** and **Fig 8A-D**). This is likely due to the different function of the lysosomal compartment in these tissues: in salivary gland 2h bpf, most of the lysosomes are fusing with secretory granules (SGs) whose content is delivered for secretion, while lysosomes in brain do not have such an extensively secretory function. Hence, we conclude that ANK-PATs have similar function in both salivary gland and adult brain regarding their acidification and activity, while changes in the morphology of lysosomes are more dependent on the tissue type and function.

3. In Fig1., the nuclei seem to be imaged at different optical depths in the various genotypes, which could create an artifactual difference in appearance. Please show all samples at the same z-plane so that layer-dependent variation is excluded.

Thank you for this useful suggestion. We have replaced the images in **Fig 1B** and also in **Fig 1E, F** (formerly the **F** and **G** respectively) panels with other ones from the same experiments. Now all panels show images that were acquired from the same Z-plane, however, as the penetration of DAPI nuclear staining was highly variable in this tissue, we removed it from almost all images, to avoid any misleading interpretations. We believe the data presented in each panels in **Fig 1** looks much more consistent and comparable.

4. In Fig 1, the number and size of Sgs3-dsRed granules are altered in Hip14 and Patsas RNAi. It remains unclear whether these changes reflect increased biogenesis or impaired maturation. Additional quantification would help clarify this point.

Thank you for this comment. We have quantified the number and size of Sgs3-dsRed granules and we found that the loss of both Patsas or Hip14 significantly decreased the size secretory granules (see **Fig 1I**) while their number did not alter significantly (see **Fig 1J**) in white prepupal (wpp) stage. Additional experiments in younger larvae (2h bpf) revealed that ANK PAT-deficient cells are defective for SG-lysosome fusion and also show SGs with decreased size, without any change in their number (see **Fig 2A-G**). In our earlier studies we demonstrated that lysosome fusion and subsequent acidification of SGs is critical for their maturation (PMID: 38331993), moreover in this current research we showed that overexpression of Hip14 is accelerating SG-lysosome fusion (see **Fig 3D** and **4D**) without significantly affecting the mean number of SGs (**Fig 3I**). Considering these findings together with no changes in SG numbers, our data rather suggest that ANK PATs – through promoting SG-lysosome fusions – are regulating SG maturation (at 2h bpf) or crinosome formation (in prepupae) instead of SG biogenesis. We also included a new summary figure (see **Fig 10**) about our suggested model how ANK-PATs are affecting SG-lysosome fusion and SG maturation and extensively discussed this in the Discussion chapter as the following:

*“In our work, we made significant efforts to find out how modulation of ANK PATs affects lysosomal maturation and fusion by assaying the localization of two different lysosomal enzymes. As a result, we demonstrated that loss of either Patsas or Hip14 leads to a significant decrease in fusion between maturing glue granules and Arl8-positive lysosomes and the LysoTracker-positive acidic compartment, resulting in reduction in size of LysoTracker positive structures, likely representing fusion incompatible lysosomes with a decreased acidification capacity. Moreover, we also observed the decline in size of Cathepsin L-positive lysosomes, decreased lysosomal hydrolytic activity by MagicRed and impaired delivery of lysosomal AcPase to maturing glue granules in ANK PAT deficient secretory cells. Vice versa, the overexpression of Hip14 resulted in the expansion of LysoTracker and Cathepsin L-positive structures, which likely represent premature crinosomes that were also positive for AcPase. These findings suggest that the activity of Golgi resident ANK PATs may serve as a rate-limiting factor in lysosome maturation, function and their fusion with SGs (Fig 10).”*

5. In Fig2 B-C, Patsas RNAi appears to decrease, whereas HIP14 RNAi increases, Sgs3-dsRed granule number. Please clarify whether this divergent pattern reflects differences in knock-down efficiency or distinct roles of the two PATs; a double knock-down or epistasis test would be informative.
Thank you for this comment. Now we quantified the SG size and SG numbers in these experiments, and we found that in line with our findings in white prepupal stage (see **Fig 1A-C, I, J**), the SG size significantly decreased in both Patsas and Hip14 RNAis, while SG number did not show significant alterations (**Fig 2F, G**). To avoid any discrepancy between the image and the statistics we changed the image in **B** panel of **Fig 2** to another one (from another animal of the same experiment) that fits better to the quantified data and represents better the Patsas RNAi phenotype. Moreover, we carried out a new experiment on Patsas, Hip14 double RNAis too, which showed the same phenotype (see **Fig 2D-G**) as we experienced in single RNAis, which further suggests that these PATs are acting in the same pathway. We also extensively discussed the possible scenarios of the hierarchical relationship between Hip14 and Patsas in the Discussion chapter as follows:

*“**One interesting finding of our work is that the loss of Patsas or Hip14 alone is enough to cause a similar perturbation in post-Golgi trafficking and lysosomal fusions. Our epistasis analysis where Patsas and Hip14 double deficient cells did not showed further perturbation in crinophagic flux than single knock downs did, suggests that these enzymes act non-redundantly. On the other hand, as ectopic expression of Hip14 could rescue both the lack of Patsas or Hip14 it rather suggests that these enzymes may act in the same pathway and likely on the same substrates, but Hip14 is functionally downstream of Patsas. An explanation for this could be that the activity of Hip14 and Patsas is closely related, as has been observed between Hip14 and other DHHC enzymes [13, 60, 61]. For example one of the enzymes binds substrates stronger, but the other performs the palmitoylation more efficiently, as suggested for mammalian Golgi PATs [13, 62, 63]. However, we also cannot rule out an alternative, although less likely explanation that Golgi has such a high demand of PAT activity [13], so the cell cannot tolerate the lack of either enzymes. Further research should be conducted to uncover what the actual relationship is between the two ANK PATs’ activities and substrate preference.”*

6. In Fig3., only vesicle size is reported. Including LysoTracker-positive vesicle counts (and mean intensity per cell) would reveal whether vesicle abundance, not just enlargement, is affected.

Thank you for your suggestion. We carried out the requested analyses and we found that LysoTracker Red intensity was significantly increased in UAS-Hip14 cells compared to the controls (see **Supplementary** **Figure S3G**). As other Reviewer’s comment (please see our response for **comment 6 of Reviewer 2**) also requested to revise and further characterize LysoTracker data, we carried out a new experiment with LysoTracker deepRed (LTdR) on same-aged larvae (2h bpf). This new experiment revealed that the majority of enlarged LTdR structures in control and Hip14 overexpressing cells are also positive for Sgs3-dsRed reporter (see **Fig 3A, D**), indicating that these highly acidic structures mostly represent mature SGs or premature crinosomes. In line with the LTR data, the LTdR intensity was significantly increased in Hip14 overexpressing cells compared to the controls (**Fig 3G**), while the number of the structures did not change (**Fig 3F**). However, in this new experiment we did not detect significant increase in the mean area of LTdR structures in UAS-Hip14 (**Fig 3E**). As the large acidic organelles mostly represent mature SGs or premature crinosomes in controls and UAS-Hip14 cells, while Hip14 overexpression did not increase the SG size (**Fig 3H**), we think that our LTdR data is representing better the real size of acidic organelles. The discrepancy between LTR and LTdR structure sizes between UAS-Hip14 and control samples, prompted us to reevaluate our LTR experiments, and we found out that there is actually much more LTR positive structures in control tissues, but they stained less intensively with LTR than they did in Hip14 overexpressing tissues. Thereby, we repeated the quantification of LTR structure size data using a lower-set threshold, and this new quantification resulted in LTR data that is fully consistent with LTdR results (**Fig 3E-G** and **Supplementary Figure S3E-G**). However, as LTdR staining gave much better-quality images and data, we decided to place it as main figure (as new **Fig 3**), and the LTR data was moved to Supplementary Material (now in **Supplementary Figure S3**). Please note we also changed the A panel of **Supplementary Figure S3** (form another animal of the same experiment), that represents better the LTR phenotype of control cells.

7. In Fig 6, several relevant control groups appear to be missing. First, the Rab2 constitutively active condition in HIP14 KD is tested, but the Rab2 dominant-negative or Rab2 WT only are not shown—this raises the question of whether granule phenotypes are driven by Rab2 activity level alone. Second, all data are from overexpression conditions; testing Rab2 CA in a rab2 mutant background would clarify whether the effect is specific and sufficient.
Finally, in Fig 6A–D, Sgs3-RFP granule number changes are observed, but their biological significance (increased synthesis vs. blocked clearance) remains unclear and should be further discussed or quantified.

Thank you for these useful suggestions. We carried out new experiments by overexpressing the wild type form of Rab2 (Rab2[WT]) in control background, and it did not affect the size of Cathepsin L positive structures compared to the control and also could not compensate for the decreased Cathepsin L structure size phenotypes of Patsas or Hip14 deficient backgrounds (see these in **Supplementary Figure S7A-D, H**). These findings indicate that the observed increase in lysosome size is indeed dependent on the activity of Rab2. We also found that knocking down Rab2 in a Rab2[Q65L] overexpressing background can also critically decrease the size of Cathepsin L structures (**Fig for Reviewers 1**).

We quantified the number and size of Sgs3-dsRed granules in Rab2[Q65L] cells, and we found that hyperactivation of Rab2 alone is enough to decrease the mean size and increase the amount of SGs (see in **Figure 7F, G**). This significant increase in SG number suggests that Rab2[Q65L] may not only promotes SG-lysosome fusion but may also support SG biogenesis (likely through Rab2’s function in promoting Golgi exit trafficking processes (PMID: 33822845)), which is a striking difference compared to Hip14 overexpression conditions (**Fig 3H, I**).

Hence, our data does not clearly support that Rab2 is acting in an ANK-PAT dependent manner (and we did not state this in the text either). Our findings rather suggest that promoting lysosome fusions by hyperactivation of Rab2 can partially compensate for but not fully rescue the lysosomal phenotype upon the loss of Patsas and Hip14.

**Fig for Reviewers 1. The size of the Cathepsin L-positive compartment depends on Rab2 activity.** The Cathepsin L-positive structures are smaller in Rab2 deficient salivary gland cells, even upon the overexpression of the constitutively active (Rab2[Q65L]) form of the protein. Insets show 2x magnification of the outlined area, split into channels. Scale bar represents 20 μm. M: merged, CL: Cathepsin L, S3R: Sgs3-dsRed, 2h bpf: 2 hours before puparium formation.

8. In Fig 6G., The red channel is severely smeared/over-saturated, obscuring structural detail.

Thank you very much for pointing out this lower-quality image. We have now replaced this panel (can be seen now as **Fig 7I**) with another image from the same experiment, where the structures look much sharper.

9. In Fig 7, control group flies show reduced climbing ability at 14 d and 21 d, raising concerns about the assay design or interpretation. Please clarify the cause of the poor performance in the control group—whether it relates to the specific genotype used, handling conditions, or assay settings—and consider replacing the control genotype if necessary.

Thank you very much for your suggestion. We repeated the entire experiment in both sexes with both the former and new controls and extended the experiment by adding other genotypes (Rab2[Q65L] alone and Patsas RNAi) (see the new experiments in **Fig 9** and **Supplementary Fig S8**). Our new control, where Appl-gal4; tub-Gal80^TS^ line was crossed with a control RNAi line, showed a much more stable climbing performance, and this time we experienced the same with the former control (crossed with W1118). Now the difference between the controls and neuron-specific Hip14 RNAi became more robust, and it was still rescued by the co-expression of constitutively active Rab2. Importantly, we also detected a decrease in the climbing performance of Patsas RNAi. The former poor performance of the control group was likely caused by a mold/bacterial infection problem that we face from time to time.

In line with these, please note we removed the former climbing data (**former Fig 7D**) and replaced it with the new more robust ones (**Fig 9** and **Supplementary Fig S8**).

10. To confirm that behavioural rescue is lysosome-dependent, pharmacological inhibition (e.g., chloroquine or bafilomycin) could be applied to test whether the rescue is abolished.

Thank you for this comment. We attempted to carry out a climbing experiment on Hip14 RNAi and Rab2[Q65L]; Hip14 RNAi flies raised on 50 nM bafilomycin supplemented media. Although, we saw some decline in the climbing ability of bafilomycin treated flies that was specific for Rab2[Q65L]; Hip14 RNAi, but not the Hip14 RNAi flies, these differences were not significant (however in some cases it was close to it) and this was only visible for shorter (20 sec) climbing times (**Fig for reviewers 2A, B**). We also tested whether bafilomycin efficiently inhibited the lysosomal degradation in the heads of control adults by testing the levels of Ref(2)p (the Drosophila homolog of p62), a protein that is specifically delivered for degradation in the lysosomes. In western blot we found weak but visible increase in Ref(2)p levels upon bafilomycin treatment compared to the loading control (**Fig for reviewers 2C)**. Hence, these results suggest that the concentration of bafilomycin in fly food, that we previously used successfully in other tissues (PMID: 35672498), only slightly decreases lysosomal degradation in control adult heads. However, considering that Rab2[Q65L] so effectively promotes lysosome fusions it is also possible that this drug feeding technique is insufficient to inhibit this and the used method may not be robust enough to test whether the rescue effect of Rab2[Q65L] on the climbing ability of Hip14 RNAi is due to increased lysosomal fusions.

**Fig for Reviewers 2**. **Pharmacological inhibition of lysosomal function by bafilomicyn A1 slightly, but not significantly decreases the climbing ability of UAS Rab2[Q65L]; Hip14 RNAi flies. (A)** The bafilomicyn treatment does not affect the climbing ability of female **(A)** and male **(B)** flies lacking Hip14, but a non-significant decrease in the motor performance can be noticed in 14-day-old UAS-YFP-Rab2[Q65L]; Hip14 RNAi flies. Black dots represent one measurement, circles indicate outliers, boxes represent the typical 50% of the climbed adults, the lines show the median and whiskers represent the upper and lower quartiles, n=20 flies per genotype. **(C)** The bafilomicyn treatment just minimally increased the level of Ref(2)P autophagic substrate in isolated control head samples, which suggests that the applied bafilomicyn concentration or the administration method is only moderately effective.

11. In the adult brain, Changes in Cathepsin L puncta size alone are not sufficient to establish lysosomal dysfunction. Co-staining with LysoTracker and Lamp1, quantifying Cat L puncta number, and measuring Magic-Red Cat L activity would substantiate the link to lysosome function.

Thank you very much for this comment. We further analyzed the morphology of the lysosomal compartment in the brain. First, we repeated the Cathepsin L staining on one-week older flies (day 21) – which showed stronger climbing defect (**Fig 9** and **Supplementary Fig S8**) –, extended the experiment for Patsas RNAi, too, and also quantified the number of the Cathepsin L punctae (**Fig 8A-E**). In this age group, the phenotype was much more obvious, so we replaced the previous data (from day 14, the **A-C** panels of **former Fig7**) with these. We also carried out LysoTracker Red staining on brains of day 21 flies that also express Lamp1-GFP reporter. In line with our previous findings with Cathepsin L, we observed that the structures that are positive for these markers are significantly larger in size in Hip14 RNAi and Patsas brains (**Fig 8F-L**). Most importantly, we also observed that GFP-Lamp1 structures showed extensive colocalization with LTR in Patsas and Hip14 RNAi but not in control brains (**Fig 8F-H**), a phenotype that was also experienced in salivary glands 2h bpf (**Supplementary Fig S3A-C**). We believe that this data more robustly suggests that lysosome function and morphology are severely perturbed in ANK PAT deficient brains.

12. In Fig 7, the Patsas RNAi group and the Rab2 mutant-only group are not clearly examined in the adult brain. Please clarify whether these genotypes were tested, and if not, provide an explanation or include the relevant data to complete the comparison

Thank you for this comment. As we mentioned at **comment 10**, we repeated the climbing experiment and this time we tested the effect of Patsas RNAi and Rab2[Q65L] overexpression (see in **Fig 9** and **Supplementary Fig S8**). Additionally, we also tested the effect of Patsas RNAi on the lysosome morphology and function (Cathepsin L and Lamp1-GFP+LTR stainings) in the brain (please see our **response for your comment 11**), which is now included in **Fig 8**.

Minor
1. In Line 346, Figure 7D is not referenced in the text.

Thank you for this comment. This has been corrected.

**Reviewer #2:** In the present study Szenci et al., the authors investigated the role of Golgi-resident ankyrin repeat-containing palmitoyl transferases (PATs), Hip14 and Patsas (Drosophila homolog of HIP14L), using Drosophila larval salivary gland. The study examined their roles in secretory and lysosomal membrane trafficking. The authos found that these PAT enzymes equally contribute to the proper maturation and crinophagic degradation of glue secretory granules by mediating their fusion with the endo-lysosomal compartment. They are also required for lysosomal acidification and biosynthetic transport of the lysosomal hydrolase CathL. Hip14 is also essential for proper lysosome formation and neuronal function in adult brains. Over-activation of lysosomal biosynthetic transport and lysosomal fusions by the expression of the constitutively active form of Rab2 could compensate for the lysosomal dysfunction caused by the loss of Patsas or Hip14 both in larval salivary glands and neurons. These findings are interesting, yet the results from the images can conclude only as much and a lot of claims in the paper are over-interpreted. A lot of terms such as “lysosome biogenesis” or “lysosome maturation” are carelessly used in the paper, which needs to be modified. For all Figures, statistical analyses are overly simple, a full and more complete analysis will help to dissect the difference better. Figure legends are poorly written. A lot of essential information were not included, misleading readers. I listed some of the major and minor concerns below:

1. Figure 1: when assessing the crinophagic flux, the statistical analysis seems rather simple. A full and more complete analysis will help to understand more in-depth on the efficiency of crinophagy. What about the number of puncta? The percentage of different type of puncta over the total number? Do PAT expression restore the defect?

Thank you very much for your suggestion. We quantified the number and size of SGs regarding the flux experiments and found that Hip14 and Patsas RNAi tissues do not contain significantly more SGs, but their size was smaller (**Fig 1I, J**). We also replaced the image in **Fig 1B** panel to another one that aligns better to the z-plane and thereby more comparable with panels of **Fig 1A** and **C**. We also quantified the ratio of the yellow (Sgs3-GFP and Sgs3-dsRed double positive) SGs among the total SGs+crinosomes (positive for at least Sgs3-dsRed). This analysis clearly pointed out that the ratio of non-acidified and non-degrading (GFP and dsRed double positive) SGs remarkably increased in Hip14 and Patsas deficient samples compared to the control (see **Fig 1G**). This finding clearly indicates the perturbed crinophagic flux in these tissues. Importantly, repeating the flux experiments with independent RNAi lines or Patsas, Hip14 double knock-down resulted in the same defect in crinophagic flux (**Supplementary Fig S1A-D**, **G**) (please see also our response for **comments 1 and 4. of Reviewer 1**). Finally, we carried out rescue experiments in salivary glands in which we simultaneously overexpressed a Hip14WT transgene together with Patsas RNAi or Hip14 RNAi (see **Supplementary Fig S1E-G**). Importantly, overexpression of Hip14 fully restored the crinophagic flux in Patsas RNAi and Hip14 RNAi tissues.

2. Figure 1: what’s PP on the upper right corner? What’s the label S3R? No enlarged insets for A-C? Figure legends need to be more clear otherwise it’s hard to follow what the authors are trying to say – all legends.

Thank you for this comment. We are sorry for this confusing practice. We have now resolved all abbreviations that were used in the figures in every figure legend (for example: PP: prepupa; S3R: Sgs3-dsRed). We also included enlarged insets for **Fig1 A-C**.

3. Crinophagic degradation: is it specific to sgs3 or also applied to other glue proteins? Are there other proteins that can be used as examples to generalize the principle?

Thank you very much for this comment. The composition of the glue material that is secreted by the salivary gland is very complex. According to flybase.org there is at least 8 different glue proteins (https://flybase.org/reports/FBgg0001189.html), and Sgs3 one of the most prominent among these. The availability of both GFP and dsRed tagged reporters and their high-level and tissue-specific expression made Sgs3 a useful and well-established reporter for studying the post-Golgi trafficking of SGs (e.g., PMID: 22791894, PMID: 38285943). Moreover, these reporters are well-established tools for studying crinophagy and crinophagic flux, and they have been used for this purpose in many papers (PMID: 29066608, PMID: 36353974, PMID: 36600084, PMID: 36306596, PMID: 38331993). As it was shown before that multiple mucin type Sgs proteins are involved in restructuring of SG content (PMID: 36252017), suggesting that they are present in SG lumen, hence we can safely assume that these other glue proteins are also subjects of crinophagic degradation, however to our knowledge there is no actual data about this in the literature.

4. According to the information in the introduction (lines 87-93), it seems like the maturation of granules involves homotypic fusion, progressive acidification, events based on granules themselves and fusion with lysosomes. Thus, it might not be feasible to analyze the maturation of granules solely based on the fusion with lysosomes.

Thank you very much for this comment. We agree that maturation of SGs is a much more complex process and cannot be reduced to a simple fusion event with lysosomes. Thereby, we also quantified the number and size of Sgs3-dsRed positive maturing SGs in salivary glands from larva 2 hours before puparium formation (2h bpf) (see **Fig 2F, G**). Importantly, we found that the lack of Hip14 or Patsas significantly reduced SG size, while their number did not change significantly. Although these findings may raise the possibility that ANK-PATs are required for homotypic fusion, if this would be the case we would expect that the overactivation of these PATs should increase the mean SG size. Importantly, we found that overexpression of Hip14 also decreased the mean SG size but not affected their numbers significantly (**Fig 3H, I**), likely because of upregulation of SG-lysosome heterotypic fusion, and premature crinosome formation (**Fig 4D**). Taking these together, our findings suggest that ANK-PATs are mostly required for promoting SG-lysosome heterotypic fusions and do not play a major role in SG-SG homotypic fusion.

5. Figure 1&2: why is forming a ring around the granule indicating fusion? The authors seem to suggest so for both endosomes and lysosomes.

Thank you for this comment. Nascent SGs are devoid of endosome (like PI3P membrane lipid reporter GFP-FYVE) and lysosome (GFP-Arl8) membrane markers (both are associated to the cytoplasmic surface of these organelles), and they will acquire these as a consequence of fusion with these organelles. In our earlier study (PMID: 38331993), we have shown that the presence of these markers on Sgs3 positive granules is dependent on SNARE-mediated fusion with Arl8 and GFP-FYVE positive lysosomes and endosomes respectively. Since Sgs3-dsRed is present in SG or crinosome lumen, while both GFP-FYVE and GFP-Arl8 are localized to the membranes, the appearance of GFP-FYVE or GFP-Arl8 rings around the Sgs3-dsRed puncta clearly represents SGs that have undergone a membrane fusion event with endosomes or lysosomes.

6. Figure 3: the identity of the LTR-positive acidic organelles need to be verified. using other markers? LTR labels all acidic organelles including granules

Thank you very much for your suggestion. Previously we described (PMID: 38331993; PMID: 36306596) that fusion with different lysosomal subpopulations and subsequent acidification is critical for SG maturation at age 2h bpf and also for crinosome formation at white prepupal stage. Considering that the size of the large acidic structures was in the range of SG or crinosome size, we carried out a new experiment with Sgs3-dsRed and LysoTracker deep Red (LTdR) and we found extensive colocalization between these two markers in control and Hip14 overexpressing cells (**Fig 3A, D**). This suggests that these large acidic organelles represent SG derivants (mature SGs and crinosomes) that have already undergone heterotypic fusion with lysosomes. This is further supported by EM analysis shown in **Fig 4A, D**, where control salivary gland cells contain mostly mature SGs and only a few crinosomes, while premature crinosomes appear in Hip14 overexpressing cells.

7. Figure 3: the size of LTR organelles does not seem to expand upon Hip overexpression, compared to control in the images. The representative images and the statistics do not correlate.

Thank you for pointing out this discrepancy. We agree that the difference between the control and Hip14 overexpression is not visible. We replaced the **A** panel with another control image (can be found now as **Supplementary Figure S3A** that corresponds to the **former Fig 3A**), which was acquired in the same experiment. Now we think that the representative image fits better to the statistics. Importantly, we also carried out LTdR stainings (can be found in **Fig 3A-G**) (please see our response for the previous comment), which promted us to reevaluate the original LTR data, by using another thresholding method in image analysis (please see more detail in or response for **comment 6. of Reviewer 1**). This re-quantified LTR data showed that the size of LTR positive structures (although they stained more intensively) in Hip14 overexpressing cells is not significantly differing from controls. This data is now more consistent with the LTR microscopy data and also with LTdR data. Please also note that we moved LTR data to the supplementary material (can be found as **Supplementary Figure S3**), while the new LTdR experiments are presented in the **new Fig 3**.

8. Figure 4: EM images show supersized granules – are them comparable with the ones in Figures 1-3?What does the scale bar indicate? Same or different in these figures? These information need to be present in the legends, otherwise it is rather hard to follow.

Thank you for this comment. The scale bars are representing 1 um for each panels in **Fig4**, while in Figs 1-3 the scale bars represents 20 um. Based on EM data from **Fig 4A** the diameter of SGs are spanning in a range of 2-6 um, consistent with the estimated sizes of SGs from the fluorescent images in Figs 1-3. So, we respectfully think that the SGs are not oversized in **Fig 4A**, **B** and **D**, the difference may only be due to different magnifications. However, we agree that **Fig 4C** really represents an oversized granule, which is not consistent with our statistics from same aged salivary glands (**Fig 2E**) showing that loss of Hip14 leads to decreased SG size. We chose to show that particular SG (in the **former C panel of Fig 4**) because of the large number of unfused multilamellar lysosomes that are next to it. However, we agree that showing such oversized SG may cause some confusion for the reader. Thereby, we now replaced this image in this panel (**Fig 4C**) with another one which represents better the SG size that is typical for Hip14 RNAi cells. We also included the following sentence into the caption of the figure:

*“Scale bars represent 1 µm on each panel.”*

Additionally we now clearly described the size of the scale bars in each figure captions.

9. Figure 5: analyzing the sorting and transport of a lysosome hydrolase does not equal to analyze lysosome biogenesis. There is a huge difference. The authors use lysosome biogenesis but no size or area of the lysosomes were measured throughout the study. Either the term needs to be corrected or the authors need to perform more experiment to clarify whether the lysosome biogenesis is really affected by PATs.

Thank you very much for this useful suggestion. We agree that in addition to the delivery of the hydrolases to the lysosomes, the number and size is also an important indicator of lysosome biogenesis. We have already shown that Cathepsin L positive lysosome sizes significantly decreased in Patsas and Hip14 deficient cells, while lysosomes were enlarged in Hip14 overexpressing cells (in **former Fig 5E** in the original version of the manuscript). Now we also quantified lysosome numbers on the same images, but we did not find any alterations in any genotype (now both quantification data can be found in **Fig 6E, F** in this current version of the manuscript). Additionally, we also carried out a Magic Red staining that reports about the hydrolytically active lysosomes (especially the activity of lysosomal hydrolase Cathepsin L) (**Fig 6G-L**). Importantly, we observed that active lysosomes were smaller in size in Hip14 or Patsas deficient salivary glands. In contrast Magic Red positive structures were significantly enlarged in Hip14 overexpressing cells. Similarly to Cathepsin L immunostaining data we did not see a significant difference in the mean number of MagicRed positive structures (quantification data about MagicRed structure size and number is now included as **Fig 6K, L**). Taking this together, our data suggests that lysosome formation, morphology and lysosomal hydrolytic activity are highly dependent on the presence of Hip14 or Patsas.

10. Figure 5: The author stated that cathL-positive compartments are fragmented. This is bizarre. From the images, one might be able to say that the cathL-positive or the Lamp1-GFP-positive lysosomes are more “puncta-like”, but no evidence has been shown that whether it’s being fragmented. Lamp1-GFP-positive puncta also exhibit different structure, more puncta-like.

Thank you for this comment. We rewrote that sentence as follows:

“*In contrast, the lack of Patsas (Fig 6B) or Hip14 (Fig 6C) equally led to the decrease in the size of Cathepsin L-positive structures, that became more puncta-like (Fig 6A-C, E)…”*

11. Figure 5: why is Hip14 overexpression causing different effect on cath-L and Lamp1-GFP?

Thank you for this question. Lamp1-GFP is a reporter where the GFP tag is facing to the lumen. The purpose of this design was that this way the GFP can be quenched and degraded in actively degrading and highly acidic lysosomes (the original description about this reporter can be read in the following publication: PMID: 16046475). As we observed that Hip14 overexpression is causing the increase the intensity of LysoTracker Red staining (likely representing increased acidification of lysosomes) (can be found in **Supplementary Figure S3G**) and the activity of lysosomal hydrolases (Magic Red) (**see Fig 6J, K**), we think that the GFP was rapidly quenched and degraded and hereby its signal is missing from these lysosomes and crinosomes (**Supplementary Figure S3D**). This is further supported by our EM experiment where observed the appearance of premature crinosomes in Hip14 overexpressing cells (see **Fig 4D**). In contrast to pH and hydrolase sensitive Lamp1-GFP reporter, Cath L is a lysosomal resident protease that is persistent even in very active lysosomes.

12. Figure 6: what's the difference between LTR and LTdR? why using two different versions throughout the study?

Thank you for this comment. Both dyes are staining acidic lysosomes and the fluorescent LysoTracker Red and LysoTracker deep Red are two related, cell permeable, vital dyes, both of which are becoming fluorescent in the acidic environment of the lysosome lumen. The two dyes are differing from each other in the emission wavelength (the maximal emission of LTR is at 590 nm, while it is 668 nm for LTdR) hence they can stain lysosomes on the red or far-red channels respectively. Originally, we used LTR to show the potential colocalization between Lamp1-GFP and LTR signal. This setup is particularly useful as LTR is already staining both very acidic and weakly acidic organelles (like late endosomes, or less acidic lysosomes around pH=6-6,5; (PMID: 36309696)), while GFP is getting fully quenched at pH=5 (PMID: 21073756). Hence dysfunctional, less acidic lysosomes will appear in yellow (with both green and red signal) as it observed in Patsas and Hip14 RNAis, in contrast to very acidic lysosomes and crinosomes that are marked by red fluorescence only (the GFP is already gone) in controls and Hip14 overexpressing cells (find this data in **Supplementary Figure S3A, D**). In contrast when we wanted to see whether mature secretory granules and crinosomes are also become acidic, we needed to use the pH resistant Sgs3-dsRed reporter (instead of the pH sensitive Sgs3-GFP), which is not compatible with LTR, and LTdR was more appropriate for this purpose (see this data in **Fig 3A-D** and **Fig7A-D**). Importantly, we also quantified the alterations in the intensity, size and number of LTR and LTdR in control, Hip14 RNAi, Patsas RNAi and Hip14 overexpressing cells, and the phenotypes changed consistently with both dyes (quantification data can be found in **Fig 3E-G** for LTdR and **Supplementary Figure S3E-G** for LTR), indicating that LTR and LTdR are working very similarly and presumably labels the same organelles in salivary gland. Please note LTR data was reevaluated and quantified again in this current version of the manuscript (see the reasoning in detail in our response for the **comment 6 of Reviewer 1**).

13. Figure 7: I don’t see a decline in the climbing ability (Figure 7D) in Hip14-RNAi-expressing animals.

Thank you very much for this comment. As Reviewer 1 also raised some concerns regarding the climbing test, we repeated the entire experiment in an extended setup (see **comment 9 of Reviewer 1**). We used two control groups now: first the appl-Gal4, tub-Gal80TS crossed with w^1118^ and in the second control we crossed the same line with UAS-GFP-RNAi. Additionally, we included two other genotypes: Patsas RNAi and Rab2[Q65L] overexpression in control background. We also carried out this experiment on both sexes and the results consistently showed that neuron-specific loss of Patsas or Hip14 leads to decreased climbing ability (find these results in **Fig 9** and **Supplementary Figure S8**). We hope this new and more extended data set will undoubtedly demonstrate that neuronal Patsas and Hip14 is critical for the integrity of neuromotor performance.

**Reviewer #3:** Post-translational modifications (PTMs) of proteins play important roles in regulating the subcellular localization, function, and stability of proteins, diversifying the spectrum of proteosome and involving in multiple biological processes. Palmitoylation is one of the PTMs, catalyzed by DHHC proteins in the ER and golgi apparatus. It has been suggested that accumulation of mutant Huntingtin proteins can reduce the activity of DHHCs like HIP14 and HIP14L (Patsas). However, how the events are triggered and the subsequent of these PATs loss of function in the pathogenesis of Huntingtin disease remains largely unclear. In this manuscript, the authors used the salivary gland (SG) of to explore the roles of HIP14 and HIP14L in the exocytosis and lysosomal degradation of glue granules, and later examined the phenotypes of HIP14/L in neurons to implicate the possible roles JIP14/L played in the pathogenesis of Huntingtin disease.
Although the authors found that HIP14/L may participate in the lysosomal degradation of un-secreted glue granules in the SG, directly or indirectly, and affect the neuronal functions, the manuscript is basically describing the phenotypes. No mechanism of the function of HIP14/L is attempted to be addressed at all. As the enzyme responsible for protein palmitoylation, how do HIP14/L function in this process?

Which substrates are they palmitoylated? Do they function simultaneously in the secretion of glue granules and lysosomal components or they involve in the degradation of non-secreted glue granules alone? Do they function similarly in neurons as they do in the SG? Considering theses important unanswered questions, I would not recommend the publication of this manuscript, unless these questions are properly addressed.

Thank you for comments and suggestions. We agree that our research did not point out one particular substrate of Hip14 and Patsas that can be responsible for the lysosomal phenotypes. However, results from mice HD models showed that accumulation of mutant Htt generally downregulates Hip14-dependent palmitoylation (described in PMID: 25849918, PMID: 20685337), raising the possibility that the lysosomal phenotype we detect in ANK-PAT deficient tissues can be caused by the defective palmitoylation of multiple targets. Hence, finding one particular or multiple targets would demand detailed analysis of the palmitoylation proteome and application of large scale advanced molecular biology techniques. We respectfully hope you may agree that this would be an entire new project itself, and it is beyond the scope of this current work.

To attempt to get some mechanistic insights in the action of ANK PATs while still keeping our work focused, we chose three proteins: Htt, PI4KII and Vamp7 SNARE for further analysis that are involved in endo-lysosomal membrane delivery, can be palmitoylated, and are potential or known targets of Hip14 or Hip14L/Patsas. We tested the loss-of-function phenotype of these three genes, and only the loss of Vamp7 caused a reduction in Cathepsin L positive lysosome size (can be found in **Supplementary Figure S5**). This suggests that Hip14 and Patsas are not affecting lysosome fusion and function through endogenous Htt or PI4KII. The role of Vamp7 in SG-lysosome fusion was shown by us before (PMID: 29066608, PMID: 38331993). We now carried out an epistasis analysis by overexpression of Vamp7 in control and Patsas or Hip14 deficient backgrounds. Importantly, Vamp7 overexpression increased the size of Cathepsin L positive structures in all genotypes (please see **Supplementary Figure S7E-H**). Together with the Rab[Q65L] (see **Fig7**) data, we now have two different treatments (overexpressing Vamp7 or Rab2CA) that can rescue or compensate for defective lysosomal membrane delivery of ANK PAT deficient cells. Hence, Vamp7 is a good candidate for being tested whether it is affecting lysosomal membrane trafficking as a substrate of Hip14 (and likely Patsas) mediated palmitoylation in a future follow up study.

In line with Your and the Reviewer 1 & 2’s claims, we quantified all data about SG size and numbers and carried out new experiments to test how ANK PATs are affecting lysosome function and Glue secretion. Our results showed that both Hip14 and Patsas are required for both lysosome formation and function and also promote their efficient fusion with SGs. SGs fuse with lysosomes for two major purpose: First, fusion with lysosomes is a part of the maturation process of SGs before secretion (at age 2h bpf), second unsecreted SGs fuse with lysosomes during crinophagy (in white prepupal stage), which leads to the degradation of unsecreted glue proteins (please see more details about this process in our recent review article: PMID: 37092295). In the previous version of the manuscript, we already showed that the lack of Hip14 or Patsas resulted in decrease in SG-lysosome fusion (can be seen now as **Fig 2A-C, Fig 4A-C**) and subsequent crinosome acidification (can be seen now as **Fig1A-C**, **Supplementary Fig S2A-C**), while overexpression of Hip14 enhanced SG-lysosome fusion (can be seen now in **Fig 4D**). We carried out new experiments for testing the role of ANK PATs in the acidification of SGs at 2h bpf by using Sgs3-dsRed reporter and LysoTracker deep Red (**Fig 3**) and their effect on lysosomal hydrolytic activity by MagicRed staining (**Fig 6 G-L**). Finally, we also performed new experiments for testing the role of these PATs in glue secretion and we found that loss of Patsas or Hip14 increased the glue secretion (see **Fig 5A-C**). In contrast, overexpression of Hip14 (**Fig 5D**) (similarly to the expression of constitutively active Rab2 in **Supplementary Fig S6**) substantially decreased glue secretion. These findings together with our EM data, where we observed premature crinosome formation aready at 2h bpf stage (**Fig 4D**), further suggests that these PATs are required for SG-lysosome fusion, not only during SG maturation but also during crinosome formation. This way we also found that ANK PATs are affecting Glue secretion likely through regulating the extent of SG-lysosome fusion, and hereby indirectly regulating SG maturation and crinosome formation. Additionally, as Hip14 overexpression promoted premature crinosome formation, our data makes it unlikely that Hip14 may be involved in the secretion of lysosomal components, and we did not see any evidence for this in our results.

We further characterized the integrity of the lysosomal compartment in ANK PAT deficient brains, by carrying out new experiments with Cathepsin L stainings on Hip14 and Patsas RNAi brains. Since loss of any of these PATs caused the most severe climbing defects at 21 days (see **Fig 9** and **Supplementary Fig S8**) this time we chose 21 days old flies for the stainings and removed the former Cathepsin L immunolabeling experiment of 14 d old flies (it was visible as **former Fig 7A-C** in the previous version of the manuscript). We observed that loss of any ANK PATs resulted in enlarged Cathepsin L positive lysosomes in the brain (**Fig 8A-E** in the current version of the manuscript). We carried out another new experiment where we assayed the morphology of acidic lysosomes by using LysoTracker Red (LTR) dye and pH-sensitive Lamp1-GFP reporter. Importantly, we found that LTR and Lamp1-GFP double-positive, less acidic, enlarged lysosomes appeared in ANK PAT deficient neurons (**Fig 8F-L**). This finding suggests that these ANK PATs are required for the maintenance of proper lysosome morphology and acidification both in salivary gland (**Fig 6A-F, Supplementary Fig S3**) and brain. Although these alterations are consistent for the loss of both Hip14 and Patsas, it is important to note that the loss of these PATs differentially alters lysosome size in brain (increases) and salivary gland (decreases), likely due to the different functions of lysosomes in these tissues. (please see also our responses to **comments 2, 9 and 11 for Reviewer 1**).

Other concerns:
1. As HIP14 and HIP14L are homolog, are they equally required or differentially in the degradation of unsecreted glue granules? If they play redundant role in this process, why depleting one of them causes defects? If they function differentially, why similar defects are observed when they are knocked down?

Thank you for this comment. We carried out Patsas and Hip14 double RNAi experiments and epistasis analysis by overexpression of Hip14 in Hip14 or Patsas silenced backgrounds (please see also our response for **comment 1 of Reviewer 2**). We found that simultaneous loss of these two ANK PATs did not cause a more severe defect in crinophagic flux than the single RNAis (see **Supplementary Fig S1D, G**), while expression of Hip14 could rescue the crinophagic flux in the lack of any ANK PATs (**Supplementary Fig S1E-G**). This data suggests that Hip14 and Patsas are acting in the same pathway, and Hip14 is acting more downstream from Patsas. The most likely explanation for this, that these PATs may act cooperatively in the same pathway. This type of relationship was already described among multiple PATs (PMID: 25253725, PMID: 20507996, PMID: 36027648). At the Golgi apparatus, one of the enzymes is more effective in substrate binding, while the others are more efficient in enzymatic activity (PMID: 25253725). We discussed this possibility in more detail the Discussion chapter (please see our response for **comment 5 of Reviewer 1**).

2. The authors showed that both the degradation of un-secreted glue granules and secretion (exocytose of lysosomal components to the PM) are affected, how are the degradation of these glue granules processed?

Thank you for this comment. Although lysosome fusions are required for SG maturation at 2h bpf especially by altering the consistency of their cargo, there is no evidence in the literature that these lysosomal components are eventually delivered for secretion (it is possible that they get recycled before secretion) and we did not state this in any versions of our manuscript. Molecular machinery of Glue granule degradation by crinophagy has been described in detail by us in multiple papers (PMID: 29066608; PMID: 36353974; PMID: 36600084; PMID: 36306596; PMID: 38331993).

3. in Figure 1, the authors indicated that the stage of development is prepupal stage, this is very confusing. The authors should indicate the defined time point of developmental stage, such as indicated in Figure 2, 2 hours before pupation.

Thank you for your suggestion. We carried out our crinophagy experiments in white prepupae, a well-defined developmental stage (based on the textbook *Atlas of Drosophila Morphology* by Sylvester Chyb & Nicolas Gompel), that lasts in the first hour of pupariation (normally begins at ~120 h of larvae kept in 25^o^C). To avoid any confusion, we changed the the term “prepupa” to “white prepupa” in the text and “wpp” in the figure panels (**Figs 1A-F, Fig 5A-D** and **Supplementary Figs S2A-F, S6A. B**), and updated this sentence of the Introduction (lines 122-124) with the following:

*“At the first hour of puparium formation (~120 hrs after egg laying, hereafter referred as white prepupal stage/wpp), most glue granules…”*

4. Again in Figure 1, as they examined the SG at the same developmental stage and the same marker (Sgs3-dsRed) is used, why there are dramatic differences in the number of Sgs3-red granules between Fig. 1A-C and Fig. 1E-G?

We are sorry for this discrepancy. As we got similar comments from other two Reviewers, we quantified the number and size of SGs in control, Patsas RNAi and Hip14 RNAi cells of white prepupae (found in **Fig 1I, J**). As Sgs3-dsRed granule sizes decreased, in both ANK PAT deficient conditions, we changed the panels of both Patsas RNAi experiments and the Hip14 RNAi panel of GFP-FYVE experiment (find these as **Fig 1B, E, F** in this current version of the manuscript) with another, more representative images from the same experiments. It is also important to note, that SG numbers showed high variance and did not altered significantly in ANK PAT deficient cells (**Fig 1J**), hence some visual differences in the images might be due to phenotypic variance only.

5. for the forced secretion/transport of glue granules by ectopic expression of constitutively active Rab2, as Rab2CA promotes the secretion of glue granules and fusion of un-secreted granules with lysosome, why there are so many glue granules observed in Rab2CA-expressing cells? The authors also found that ectopic Rab2CA overrides the defects observed in HIP14/L knockdown cells, how this happens?

Thank you very much for this comment. We have previously described that Rab2CA promotes lysosomal fusions by recruiting HOPS tethering complex to lysosomes and late endosomes (PMID: 31194677), which is responsible for every lysosomal fusion event such as SG-lysosome fusion (we also described this in this paper: PMID: 29066608). Since the core machinery of SG-lysosome membrane fusions (SNAREs, HOPS, Rab2 and Rab7) is present and the hyperactivated Rab2 recruits and stabilizes ectopic amount of on HOPS to the lysosomes, we believe that this is enough to override or compensate for the deleterious effect of ANK PAT deficiency and thereby accelerate lysosome fusions. As we also described that Rab2CA is mostly localizes to enlarged crinosomes (PMID: 29066608), it is likely that immature glue granules directly fuse with these enlarged crinosomes and cannot undergo proper maturation process. Hereby, Sgs3 positive granules will be represented by two fractions in Rab2CA cells (see **Fig 7B**): the enlarged crinosomes, and the small immature SGs that have not fused with lysosomes or crinosomes yet. Importantly, we also quantified the SG numbers in Rab2CA background and found that their number was significantly increased (see **Fig 7G**), which raises the possibility that Rab2CA may also promote SG formation, which could be explained by the known role of Rab2 in promoting Golgi exit trafficking (PMID: 33822845). (please also see our response for **comment 7 of Reviewer 1**).

6. the transition from the SG to the neurons/brain is not supported by the same mechanism as assumed by the authors.

Thank you for this comment. As we mentioned in the response for your general remark (please see above), we carried out new experiments by staining LTR together with Lamp1-GFP (please see **Fig 8F-L**) and also repeated the Cathepsin L staining in the brains of 21-day-old control and Patsas or Hip14 RNAi flies (see **Fig 8A-E**). Importantly, we observed the appearance of enlarged LTR and Lamp1-GFP double-positive structures, which likely represent weakly acidic, dysfunctional lysosomes, that were rarely observed in controls. Importantly, appearance of LTR and Lamp1-GFP double positive, dysfunctional lysosomes was also experienced in ANK PAT deficient salivary glands (**Supplementary Figure S3B, C**), indicating that the loss of Patsas or Hip14 similarly perturbs lysosome function and acidification in both tissues. Although the size and morphology of LTR and Cathepsin L positive structures altered in the opposite direction in ANK PAT deficient salivary glands and brain, this is more likely due to the different role of lysosomal compartment in the cellular physiology of secretory cells of the salivary gland (lysosomes are mostly supporting SG maturation or crinophagy here) and neurons (lysosomes does not exclusively required for secretion here). Please see also our responses to **comments 2, 9 and 11 for Reviewer 1**.
